# Traditional grain alcohol (*bai jiu*, 白酒) production and use in rural central China: implications for public health

**DOI:** 10.1186/s12889-015-2594-4

**Published:** 2015-12-19

**Authors:** Ling Qian, Ian M. Newman, Wen Xiong, Yanyu Feng

**Affiliations:** Chinese Center for Health Education, People’s Republic of China, Beijing, People’s Republic of China; Department of Educational Psychology, University of Nebraska–Lincoln, Lincoln, NE USA; Heng-Gou Town Central Health Center, Xianning, Hubei People’s Republic of China; Health Inspection Institute, Health and Family Planning Commission of Chaoyang District, Beijing, People’s Republic of China

**Keywords:** Unrecorded alcohol, Artisanal, Homemade, Informal, Noncommercial, Traditional, 白酒, China, Spirits, Observation, Interview

## Abstract

**Background:**

An estimated 25 % of the alcohol consumed in China is traditional unrecorded alcohol produced and distributed informally. Consequently there is concern about its safety and its contribution to public health risk. Little has been written about this type of alcohol in China.

**Methods:**

Researchers observed the manufacture of traditional *bai jiu* in a rural area of Hubei Province, Central China. Two hundred fifty-nine individuals were interviewed, either individually or in small groups, about their use of and attitudes toward *bai jiu*. Individuals who made or sold *bai jiu* were interviewed about local production, distribution, and sale. Key community leaders were asked about risks from local *bai jiu* production, sale, and use.

**Results:**

All of the *bai jiu* makers followed the same basic traditional procedure. Most had learned their craft from a family member or by apprenticeship, and their product was sold to neighbors or nearby villagers. *Bai jiu* makers typically had a business license and a health certificate. The shops that bought and sold traditional *bai jiu* were family-run businesses that sold both traditional *bai jiu* and commercial alcohol to clientele within a close social network. Alcohol (all types) was consumed by 79.9 % of interviewed villagers (89.7 % of males, 50.0 % of females). Of the 207 drinkers in the sample, 72.9 % drank *bai jiu*, 59.4 % drank beer, and 22.7 % drank commercial spirits. *Bai jiu* was most often consumed at mealtimes. *Bai jiu* drinkers believed moderate drinking was healthy and that drinking improved the social atmosphere, and about one-third of them believed drinking too much could result in quarrels and family problems. The *bai jiu* business provided two sources of income for makers because spent grain from the distillation process could be fed to livestock.

**Conclusions:**

Production, sale, and use of traditional *bai jiu* occurred within the context of local traditions, values, customs, and social networks. The data did not suggest any significant issues related to contamination. Drinking patterns were similar to those found in other studies of alcohol use in China. *Bai jiu* was sold mainly to middle-aged or older men, suggesting *bai jiu* production and use could gradually disappear without intervention.

## Background

The World Health Organization (WHO) estimated that 25 % of alcohol consumed in China is unrecorded [1], which WHO defines as alcohol that is not taxed and is produced, distributed, and sold outside formal channels. This includes homemade or informally produced alcohol (legal or illegal), smuggled alcohol, alcohol intended for industrial or medical uses but used in beverages, alcohol obtained through cross-border shopping, and alcohol consumed by tourists [2].

The WHO estimated that in China between 2003 and 2005, 1.7 l of the 5.9 l of pure alcohol consumed by persons aged 15 years and older was unrecorded alcohol [3]. Between 2008 and 2010, estimated total consumption increased to 6.7 l, but unrecorded alcohol remained at 1.7 l [1]. Despite its prevalence, there are no descriptions of traditional *bai jiu* production and use in China, including the variables discussed here. Assessments of the contribution of alcohol to the global burden of disease often have no option but to rely mainly on recorded alcohol sales data; therefore, it is necessary to develop a better understanding of unrecorded alcohol production, consumption, and risks, especially in locations where unrecorded alcohol is legal and accessible. A systematic review of published papers on the epidemiology and composition of unrecorded alcohol has noted “a relative dearth of information in China” (p. 884) [4].

In 2012, it was estimated that 5.9 % (3.3 million) of all deaths worldwide and 5.1 % of disability-adjusted life years (DALYs) were attributable to alcohol consumption [5]. The percentage of all DALYs attributable to alcohol for Chinese males was the second highest among 10 large countries, and was the fifth of ten countries for females [6]. In 2010, an estimated 9.1 % of the male population and 0.2 % of the female population in China had experienced alcohol use disorders and alcohol dependence in the previous 12 months [1]. The total contribution of unrecorded alcohol, and traditional *bai jiu* in particular, to the burden of disease in China is unknown. Data on alcohol and traffic crashes in China is limited. A survey in two southern cities reported 4.5–4.6 % of the drivers with blood alcohol concentrations (BACs) over the legal limit (20 mg/100 ml) and reported 20 % of traffic crashes were alcohol related [7].

The International Center for Alcohol Policies (now the International Alliance for Responsible Drinking) has outlined that the indirect costs of unrecorded alcohol in Europe, Africa, Central and South America, and Asia come from adulteration, counterfeiting, involvement of organized crime, low prices, and high alcohol by volume (ABV). They note that unrecorded alcohol deprives governments of tax revenue. Because unrecorded alcohol typically has higher alcohol content than recorded alcohol and sells at a lower price, it has both a market advantage and an attraction for vulnerable populations [8]. Because the International Alliance for Responsible Drinking is funded by the world’s leading alcohol producers, its estimates of the dangers and the social costs of unrecorded alcohol have been seen as self-serving and aimed at encouraging policies to restrict and eliminate unrecorded alcohol from the alcohol marketplace [9, 10].

Rehm and colleagues [4, 11] acknowledged the difficulties in linking unrecorded alcohol use to specific health outcomes and concluded that ethanol presents the greatest risk to public health. In a more recent paper, they suggest that the contribution of unrecorded alcohol to cirrhosis may have been overstated because of the overlooked effects of heavy episodic drinking, socioeconomic status, and health [12].

### Study objectives

This study had two objectives: 1) to observe local makers distilling traditional grain alcohol (*bai jiu*) in a rural area in central China and 2) to conduct interviews with *bai jiu* makers, sellers, consumers, and key community members regarding production levels, methods of distribution, pricing, patterns of use, and health risks of *bai jiu*. Because both the production and consumption of traditional *bai jiu* occurred within the same geographic area, we felt it important to present the findings of both the observation study and the interviews in one document.

This paper focuses specifically on one form of unrecorded alcohol in China: locally and traditionally produced distilled grain alcohol, referred to as *bai jiu* (白酒). The word ‘*bai jiu*’ is used to refer to all distilled alcohol, not just unrecorded alcohol, and it is the most commonly consumed type of alcohol in China. Throughout this paper, *bai jiu* will be used to refer specifically to the unrecorded distilled grain alcohol that is locally made according to traditional methods. We will refer to other types of alcohol as commercial, recorded, or unrecorded alcohol.

### Study site: Xianning

This research was conducted in Xianning, Hubei Province, a prefecture-level city that includes one county-level city, one district, and four counties. In the rural areas, small towns are surrounded by smaller villages, and the villages are surrounded by agricultural fields. Farm families live together in the villages, within walking distance of their fields. The total area of Xianning is 9860 km^2^ and the population is approximately 2.8 million. Agriculture and forestry are the two principal occupations.

### Ethical review

The observational study was approved by the Institutional Review Board (IRB) of the University of Nebraska–Lincoln (Approval #: 20110611813 EX), which also approved use of data from individual and small group interviews (Approval #: 20110511816 EX). Individual and small group interview study was approved by the IRB of the School of Public Health, Tongji Medical College, Huazhong University of Science and Technology.

## Part 1: observation study

### Method

Two investigators travelled to rural Xianning, observed *bai jiu* production, and talked with local producers in 11 villages. The geographic location was chosen based on local reports of production patterns in this part of Central China. Traditional *bai jiu* production in this area was not illegal.

### Results

*Bai jiu* manufacture was found to occur in two different settings: permanent production facilities and temporary makeshift production sites set up by itinerant *bai jiu* makers who traveled from village to village to make *bai jiu* for individual families. The basic production process was the same in both settings.

Grain (rice, sorghum, or buckwheat) was soaked in water for approximately 24 h. Next, the grain was loaded onto a woven bamboo grid and steamed above boiling water in a kettle (pot still) for about an hour, or until the grain began to split. These kettles were round tubs, typically made of wood, although some larger kettles were made of aluminum or stainless steel. The kettles were about 3 ft deep and 3–5 ft in diameter. In the permanent production facilities, kettles rested on a concrete or brick base, with a firebox under the kettle. In the temporary production sites, the kettles were set on temporary bases of bricks and mud, most often on the sloping bank of a stream to make digging out a fire pit under the kettle more convenient and to have a source of cool water at hand.

After the grain was steamed, it was spread out on the ground about 4 in. deep to cool. When it reached a certain temperature (still warm), the yeast (*qu*) was spread over the grain, then mixed thoroughly and allowed to ferment. The characteristics of the yeast and the air temperature determined the length of the fermentation process. Makers said that the source of the yeast was critical to determining the quality of the *bai jiu*. Some makers produced their own yeast, and others purchased their yeast on the commercial market, sometimes from other provinces. About 150–250 g of yeast was required to ferment 50 kg of grain, which would yield 20–23 kg of *bai jiu*. One maker mentioned needing 350–400 g of yeast for fermentation during cold weather.

At the permanent sites, the fermenting mixture was covered and held for a period ranging from hours to as long as a day. Next, the fermenting grain was shoveled into either aboveground concrete fermentation boxes or into fermentation pits dug below ground level and lined with concrete. These boxes and pits were typically 4–5 ft deep and 4–5 ft wide. At some locations, boxes and/or pits were larger. In the box or the pit, the fermenting grain was covered with empty grain bags, tarpaulins, and/or plastic, and sometimes covered over with grain chaff to retain the heat generated by the fermentation. Depending upon the activeness of the yeast and the local temperature, this stage of fermentation lasted for 7–15 days. Cooler temperatures required longer periods. One maker referenced fermentation times as long as 18 days in winter.

At the temporary sites, the itinerant *bai jiu* makers usually wrapped the warm grain-yeast mixture in tarpaulins that were laid on top of a layer of grain chaff to insulate it from the ground. The tarpaulins were then covered with a thick layer of chaff. This grain was fermented inside the tarpaulin for about 24 h, then transferred to fermentation pits dug in the ground. These pits were about 3 ft deep, 2–3 ft wide, and approximately 6 ft long (depending on how much grain was to be processed). In one site where this process was observed, pits in the ground from the previous year remained, adjacent to the new pits. (It was unknown why the old pits were not reused.) The pits were lined with plastic, tarpaulins, or other available materials. The fermenting grain mixture was shoveled into the pits, covered, and the fermentation process allowed to proceed for 10–15 days. While the fermentation took place, the itinerant *bai jiu* maker usually travelled to other villages, returning to each village after 10–15 days to carry out the distillation process.

After fermentation, the grain was transferred back into the kettle (the round wooden tub described earlier) with a fire underneath. The water in the kettle was boiled to steam the fermented grain. The steam was then condensed and the distillate collected. Distillation took place in one of two ways. In the permanent facilities, a convex lid was placed on the kettle and the collected steam ducted to a condenser in which the steam was run through smaller pipes immersed in cold water to facilitate condensation. For maximum efficiency, the cold water in the condenser was replaced continuously to keep it cold. The distillate, the *bai jiu*, was collected from the end of the pipe running through the condenser.

In the makeshift itinerant sites, the kettle lid was made of metal and shaped into a shallow inverted cone with an approximately 12-inch-high lip on the concave side. The inverted cone was placed over the kettle containing the fermented grain, and the entire lid cooled by filling it with cold water. Steam, having passed through the fermented grain, reached the cold surface of the cone and condensed. The lid’s shape allowed the distillate to run to the center point of the cone, where it dripped into a wooden collection bowl and was then piped out of the kettle and collected in a separate container. Keeping the water in the lid cool enough to cause efficient distillation was critical to the quality of the *bai jiu*. Because the itinerant *bai jiu* maker did not have piped water, he continuously replaced the water in the lid with cool water from a nearby stream.

The first *bai jiu* collected from the distillation was strong and had an objectionable taste, but as the process continued, the quality of the *bai jiu* improved. Some makers discarded the first distillate; others mixed the first distillate back into the rest of the *bai jiu* to dilute its objectionable qualities. This first *bai jiu* was mostly acetaldehyde because its boiling point is lower than ethanol’s. At the end of the process, the *bai jiu* content of the distillate became too low and the distillation was discontinued. Some makers kept some of the used grain left over from the distillation process and added it to the next batch of fresh grain to be fermented, while others used the spent grain as animal feed.

## Part 2: Individual and small group interviews

### Methods

In each village, the researchers initially contacted the village leader for help finding people to participate in interviews. A total of 218 village residents responded, and participated in either individual or group interviews. The research team also contacted and interviewed nine *bai jiu* sellers, 11 *bai jiu* makers, and 21 key community members (nine physicians, six teachers, and six community leaders). In all, 259 individuals were interviewed. This sample was not randomly chosen.

A specially trained team of 10 health workers conducted interviews and group discussions with 259 individuals, either individually or in small groups, in the 11 villages where *bai jiu* production was observed. All interviews were preceded by a description of the project, followed by a declaration of willingness to participate, and an understanding that participants could leave the interview at any time. Signed consent was not feasible because many participants were functionally illiterate. Nobody declined the invitation to participate.

Participants were asked a set of core questions about demographic information, drinking practices (frequency, amount, type, and occasion/situations), beliefs about *bai jiu*, and consequences of use. Interviewers recorded the answers. Eleven *bai jiu* makers and nine *bai jiu* sellers answered the core interview questions as well as some additional questions about making and selling *bai jiu*. The *bai jiu* makers interviewed in Part 2 were not the same makers observed in Part 1. Researchers also sought out 21 key community members (local physicians, teachers and community leaders) who, in addition to answering the core interview questions, were asked specifically about their knowledge of traditional *bai jiu* (how it is made and where it is available) their experiences with the consequences of its use, the special occasions where it is used, their perceptions of local use, and perceptions of the local makers and sellers.

A field supervisor was present throughout the entire project observing interviews and checking the quality of results. At the midpoint and at the end of the interview data collection period, all interviewers met with the project director for a half-day debriefing and discussion of the findings. The data were tabulated by the Chinese authors.

### Results

#### Tabulated results from the core interview questions

All 259 individuals interviewed answered the core questions. The sample is described in Table 1. The results from the small number of female respondents precludes reliable interpretation, but their answers are reported here because this is the first known study of traditional *bai jiu* use in this area.

**Table 1:** Sample description

	Male		Female		Total	
	N	%	N	%	N	%
Gender	195	75.3	64	24.7	259	100
Married	180	92.3	61	95.3	241	93.1
Education						
Primary or less	50	25.6	25	39.1	75	29.0
Junior high school	77	39.5	29	45.3	106	40.9
Senior high school	68	34.9	10	15.6	78	30.1
Total	195	100.0	64	100.0	259	100.0

Data on age not collected

#### Drinking behavior

Individuals were classified as drinkers if they responded, “yes” to the question “Do you drink alcohol?” Of the 259 people interviewed, 207 (79.9 % of the total) were classified as drinkers (89.7 % of the males and 50.0 % of the females). Of the 207 drinkers, 82.9 % of the males and 50.0 % of the females had their last drink “today or yesterday”; 90.3 % of the males and 62.5 % of the females drank alcohol in the last week; and 96.0 % of the males and 81.3 % of the females drank alcohol within the last 2 weeks. Twenty-one percent (21.7 %) of the drinkers typically drank once per day (18.3 % of male drinkers; 40.6 % of female drinkers), and 47.8 % of the drinkers said they drank two to three times per day (54.9 % of male drinkers; 9.4 % of female drinkers).

#### Type and quantity of alcohol

Most of the drinkers drank more than one type of alcohol. Among the males, the alcohol of choice was traditional *bai jiu* (77.8 %) and beer (56.6 %). Among the females, the choice was beer (75 %) and traditional *bai jiu* (48 %). Recorded alcohols were consumed by 23.4 % of the male drinkers and 18.8 % of the female drinkers.

Beer is a common choice during hot weather, and this study was completed during the hottest months of the year. Of the people who consumed beer, 80.8 % of the males and 58.4 % of the females consumed between 500 and 1500 ml when they drank (from one to three bottles). Of the people who consumed traditional spirits, 78.5 % of the males and 83.3 % of the females drank four *liang* or less per drinking occasion. One *liang* is equal to 50 g (1.7 oz). For the people who drank recorded alcohol other than beer, 80.4 % of males and 100 % of females drank four *liang* or less per drinking occasion.

#### Time and place of drinking

Eighty percent (80.5 %) of beer drinkers reported “most likely” drinking beer at lunch, and 78.9 % at dinner. For the commercial alcohol drinkers, 85.1 % reported they drank commercial alcohol at lunch, and 87.2 % at dinner. Traditional *bai jiu* drinkers followed a different pattern: 22.2 % drank with breakfast, 80.0 % with lunch, and 94.8 % with dinner. The likeliest location for consuming beer was “at home” (67.5 %) and “with friends/relatives at dinner” (54.5 %). Commercial alcohols were likeliest drunk “with friends/relatives at dinner” (78.7 %) and less frequently “at home” (40.4 %). Traditional *bai jiu* was most likely to be drunk “at home” (84.4 %) and not so often “with friends/relatives at dinner” (47.4 %). Males were more likely to report drinking alone (66.3 %) than females (18.3 %).

#### “Appropriate” drinking and drunkenness

The majority of people interviewed (92.3 % of the males, 89.6 % of the females) believed that 1–5 *liang* was the appropriate amount to drink; but 69.7 % of the males and 21.9 % of the females said they had exceeded this amount on occasion. Despite this, only 28.6 % of the men and none of the women reported ever “being drunk.”

The majority of villagers agreed with a statement that moderate drinking was good for health (82.2 %); about half agreed with a statement that excessive drinking was bad (52.9 %). A minority (44.0 %) agreed with a statement that drinking could be addictive, and 79.5 % agreed with a statement that drinking improves the atmosphere of a social gathering, but you should not get drunk.

#### Reasons for drinking (open-ended questions)

Interviewers asked a series of open-ended questions about peoples’ reasons for drinking alcohol and recorded their responses. The four most commonly mentioned reasons for drinking alcohol were: responding to or seeking a good mood (48.5 %), dealing with a bad mood (32.4 %), socializing with friends (29.4 %), and overcoming tiredness (14.7 %).

The villagers were asked an open-ended question about “what function do you think alcohol has?” to understand further their motives for drinking. The open-ended question elicited 21 different responses. Only 10 responses were mentioned by more than 5 % of the sample, of which the most frequently mentioned were: to relieve tiredness (42.9 %), small amount of alcohol promotes good health (17.4 %), aids sleep (17.0 %), helps make friends (11.6 %) and strengthens the body (10.0 %).

The benefits of alcohol most frequently mentioned by interviewees were: making friends (45.3 %), getting energy (39.2 %), and small amount is good for health (17.6 %).

The negative impacts of drinking interviewees most frequently mentioned were: quarreling (36.7 %), family conflicts (26.5 %), bad for health/went to hospital (20.4 %), and making mistakes/being forgetful (16.3 %).

### Specific questions for *bai jiu* makers

Researchers interviewed 11 *bai jiu* makers. Nine *bai jiu* makers were male, two were female, and all were Han Chinese. Ages ranged from 23 to 65 years (<30 years = 1; 31–40 years = 3; 41–50 years = 6; and 51+ years = 1). All but the youngest were married. Nine had the equivalent of a junior high school education, one a primary education, and one a technical secondary education. Ten had learned to make *bai jiu* from other family members or by some type of apprenticeship. In other words, they had learned “on the job.” All the *bai jiu* makers we interviewed reported that only family members were involved in the production process, typically the husband and wife with occasional help from their son or daughter. Two said they worked alone. *Bai jiu* makers estimated their annual *bai jiu* production to range from 130 kg to 50 t (10 t or less = 5; 11–20 t = 3; 20–50 t = 0; more than 50 t = 2). One *bai jiu* maker claimed to be new to the business and had no production data to share.

*Bai jiu* produced from rice had an ABV ranging from 47 to 60 %, with the most popular being in the 50–55 % range. Prices ranged from 3 to 10 RMB per *jin* (approximately 0.5 l), with the best-selling *bai jiu* in the 4–5 RMB per *jin* range (≤USD0.82 per half-liter). Five makers produced *bai jiu* from sorghum with ABVs ranging from 50 to 53 % and sold it at 3.5–6 RMB per *jin* (≤USD0.97 US per half-liter). Eight makers produced *bai jiu* from buckwheat with ABVs ranging from 48 to 55 % and and prices ranging from 6 to 12 RMB, respectively, with most buckwheat *bai jiu* selling for 8–10 RMB per *jin* (≤USD1.63). Two makers produced *bai jiu* with glutinous rice with ABVs ranging from 32 to 50 %. One maker added osmanthus petals to his product and charged 5 RMB for 30 % ABV *bai jiu* and 6 RMB for 50 % ABV *bai jiu*. The 50 % ABV *bai jiu* was the most popular. For comparison, beer ranged in ABV from 3 to 4 %, and 500 ml bottles sold for between 1.5 RMB and 10 RMB (USD0.25–1.63), depending on the brand.

Ten makers determined the ABV of their product with a hydrometer, but most of them also said they judged the ABV by taste. One assessed strength of his product using the *jiuhua* method, in which he blew down the side of a reed that was touching the top of the *bai jiu* in a container and created bubbles on the top of the *bai jiu*. ABV was determined by how quickly the bubbles dissipated. One maker said that humidity at the time of production was important in the manufacturing process. He Indicated that *bai jiu* manufactured at humidity under 40 % was best.

These *bai jiu* makers were also *bai jiu* sellers. They reported that their customers were mostly middle-aged or older men with only a few younger people consuming the product. They sold their product directly from their production facility. Three *bai jiu* makers also relied on friends or small canteens to sell their product outside their production facility, three sold their product to restaurants and small stores, and five took their products to surrounding villages and towns to sell.

*Bai jiu* makers with a permanent production site typically had a business license and a health certificate. They reported occasional inspections.

### Specific questions for *bai jiu* sellers

Nine *bai jiu* sellers were interviewed in their shops. These were shops that only sold alcohol. Some shops were at the same location where the *bai jiu* was made. The interviews reported here are with *bai jiu* sellers who are not involved in making *bai jiu*.

Four of the nine sellers were female, all were Han Chinese, two were in their 30s, five in their 40s, and two in their 50s. All were married. Two had a primary education, three had a junior high school education, and four had a high school education. Four said they had been in the business for 10 years or less, three for 11–20 years, and two for more than 20 years. None of these sellers employed any assistants. Typically, the business was family run, usually with spouse, and occasionally children, assisting.

All grain *bai jiu* sold had ABVs ranging from 45 to 52 %, with the best-selling products reported to be 50 to 52 % ABV. Prices ranged from 3 to 5 RMB per *jin*. Six of the shops also sold commercially produced beer and other commercial alcohols. The beer was typically ABV 3–4 % with prices ranging from 1.5 RMB to 10 RMB for a 500-milliliter bottle. The commercial distilled alcohol products tended to have ABVs lower than the traditional *bai jiu*, and had prices ranging from 10 to 40 RMB (USD1.63–6.53). Two of the shops sold only traditional *bai jiu*.

Only two of the sellers knew that traditional *bai jiu* was made from grain and only one could describe anything about the process of fermentation and distillation. Sellers were essentially ignorant of the production process. Five of the sellers said they determined the quality by tasting and smelling the *bai jiu*. Four said they did not know how to determine the quality themselves and based their description of their products on information they gained from their customers. All they knew about the alcohol content of their products was what they had been told by the *bai jiu* maker. Typically, the sellers reported that their customers were middle-aged or older men who were local farmers. Three sellers mentioned that some of their customers were younger men.

One seller reported selling less than 100 kg per year, three sellers sold from 101 to 200 kg per year, and one sold from 401 to 500 kg per year. Four said they sold more than 500 kg per year, and up to 15,000 kg. We were later told by other community members that sellers were unlikely to report accurate estimates of the quantities sold because of uncertainty of how local officials would interpret the information.

### Specific questions for key community members

We wished to interview key community members to learn how they viewed traditional *bai jiu* and its makers, sellers, and users. Interviews of this type are new in China, especially in rural areas. Consequently, we were concerned that there would be a natural hesitancy to give candid and accurate answers to questions. To minimize this hesitancy, we identified three types of local residents for whom we thought answering questions might be less threatening: medical practitioners, teachers, and community committee members. They represented a limited, but important, segment of the community. We interviewed nine physicians, six teachers, and six community committee members (21 people). Community committees are part of the democratization process in rural China, by which villages and towns can elect local leadership from their residents. These committees serve as town/village councils that manage a range of local issues.

All 21 key community members were Chinese Han, 19 were males, and one doctor and one teacher were female. Ages ranged from 30 to 54 years (30–39 years = 7; 40–49 years = 8; and 50–59 years = 6). All 21 were married. Only three of the six teachers could identify production or sales locations. Eight of the nine physicians were aware of local *bai jiu* production sites and locations where traditional *bai jiu* was sold. All of the community committee members knew about local sales and production sites and appeared to know more about traditional *bai jiu* than the physicians or teachers. This is not surprising considering that the occupations of physicians and teachers meant the majority of their day is spent in specific locations and interacting with patients or students, while community committee members, as local politicians, visited and interacted with a broader cross-section of their communities.

All three groups reported that traditional *bai jiu* was used mostly by older men (40–50+ years old). They saw *bai jiu* manufacturing as a normal community activity. All reported that traditional *bai jiu* could be made from rice, sorghum, buckwheat, and in some cases, glutinous rice, and they knew the process involved in fermentation and distillation. Estimates of “average” amounts consumed in a drinking occasion ranged from 0.5 to 9 *liang*; physicians estimated 2.8 *liang*, teachers estimated 3.9 *liang*, and resident committee members estimated 4.1 *liang*.

There was no evidence of any negative feelings towards traditional *bai jiu* makers that could be linked to any negative values associated with its production or its use. There was a hint of suspicion towards *bai jiu* sellers who were not also the makers. Interviewees believed that these sellers had a vested interest in maintaining the quality of their product, but also might be tempted to modify the product (e.g., diluting with water) for financial gain.

There was a general consensus that traditional *bai jiu* products were safe, and many of the key community members believed them to be “more pure” than commercial products. Most of the key community members knew the typical prices of *bai jiu* and the reasons why prices varied. The key community members reported that traditional *bai jiu* was frequently used for festivals, weddings, and funerals, in part because it was “traditional,” and in part because of the lower costs of serving large numbers of people.

These key community members were also asked to describe local problems caused by alcohol in general (not just by traditional *bai jiu)*. Without exception, physicians mentioned intoxication; five physicians also mentioned fights and having to treat fight-related wounds. They commented on stomach and liver damage, and four mentioned gastric lavage. Five mentioned problems with fluid balances.

Without exception, teachers described alcohol-related problems in terms of students. Three indicated that students should not drink and the others indicated that consumption of low-alcohol beer during holidays and special celebrations was appropriate. Teachers suggested direct punishment, education, persuasion, explaining the harmful effects of alcohol, and reporting students’ bad behavior to their parents as ways to prevent alcohol-related problems. None of the community committee members mentioned any alcohol-related problems in their communities.

## Discussion

In this section, we discuss the public health implications of the data gathered. Data were obtained from observations of traditional *bai jiu* production, from interviews with users about their patterns of use, and from interviews with makers, sellers, and key community members.

### Public health implications of the production method

*Bai jiu* production was observed at two types of sites: permanent facilities and temporary facilities used by itinerant makers. Production methods in both cases were essentially the same with two exceptions. Fermentation in the permanent facilities usually occurred in boxes, typically concrete, above or set in the ground. Fermentation by itinerant makers occurred in pits dug in the ground and lined with used grain sacks, tarpaulins, or other similar materials.

Kettles were typically made of wood or aluminum or occasionally stainless steel. Wooden kettles had metal bases and lids. Small-scale production is common so there is an established and trusted supply chain for equipment. The composition of the kettle suggested little public health risk. The water was from local sources and boiled in the production process either to heat the grain initially or during fermentation. Only steam was in contact with the grain. The condenser pipes were simple with some or all of the piping made from bamboo, sometimes with a short metal section. The risk of alcohol contamination from contact with pipe and condenser material was small. There is a possibility that the empty sacks used by itinerant makers to line the fermentation pits and by permanent makers to cover concrete fermentation boxes may have once been used for pesticides or herbicides, which would introduce a potential health risk. There was also a possibility of microbes or vermin entering the grain during fermentation in either the tanks or the pits. Any consequences from this were minimized by the steam heating that occurred during the distillation phase. The final product is approximately 40 % alcohol or higher and acidic, greatly reducing the likelihood of microbes [13]. The ceramic pots used for storing *bai jiu* are a commonplace type of ceramic that has been used for hundreds of years for holding *bai jiu* or other foods, such as fermenting cabbage, that are meant for human consumption. The fuel used for the fires was local wood, and in some cases chaff from the rice or other grains was used as insulation for the fermentation process. While it is not possible to rule out risk completely in this production process, the risk from contamination seemed remote.

### Public health implications of *bai jiu* patterns of use

Heath [14] describes how social and cultural contexts influence drinking. From a Western perspective, it is difficult to imagine the social context for drinking in these rural Chinese communities. For example, villagers offered a broader range of reasons for drinking than would be typically reported in the West, and drinking solely for intoxication was not one of them. There is a longstanding belief that a small amount of alcohol daily is good for health. Similarly, in certain social situations in China, there is significant social pressure for everyone present to drink. Furthermore, compared with men, women were hesitant to give information about their drinking. People in China may not consider a drink taken at mealtime as “drinking.” They may only report alcohol drunk at special occasions (celebratory alcohol) as “drinking.” Some people in China do not count beer as alcohol. Therefore, it is likely that the self-reported estimates of drinking frequency by the men, and especially by the women, whom we interviewed are lower than their actual drinking. Reviews of alcohol use surveys in China have noted the general difficulty in gaining accurate estimates of alcohol use as well as the lack of uniform definitions for drinks, drink size, and drinking status [15, 16]. More work is needed to ensure accurate drinking pattern data.

The literature about unrecorded alcohol suggests males are the principal users and that the frequency of use was higher for unrecorded alcohol than for recorded alcohol [4]. Our data confirmed both higher use among men and greater consumption of unrecorded alcohol compared to recorded alcohol.

About 70 % of male and 21.9 % of female *bai jiu* drinkers said they occasionally exceeded the “appropriate” amount of not more than 4 *liang* of alcohol per occasion. However, only 28.6 % of the males and none of the females admitted ever “being drunk”. This suggested possible cultural controls surrounding *bai jiu* use, specifically adherence to a Confucian teaching that does not prohibit alcohol as long as one does not act in a manner that harms relationships. It is evidence for the idea that drinking comportment is affected as much by cultural controls as by the physiological effects of alcohol [17].

The *bai jiu* drinkers we interviewed were more likely than recorded alcohol drinkers to drink at meals, including the first meal of the day. *Bai jiu* was consumed frequently, commonly with meals, most often at home, and sometimes used to modify mood. About a quarter of interviewees reported negative impacts from drinking. The pattern of alcohol use found among this sample is similar to the general pattern of alcohol use described by Millwood et al. [18]. The higher ABV of *bai jiu* presents the main risk to the individual’s health, but it is difficult to estimate the contribution of *bai jiu* use to specific health conditions [4, 11] or to separate *bai jiu* use from other factors present in rural areas that contribute to diseases [19].

### Health implications of local context of production, sale and consumption

Traditional *bai jiu* makers were knowledgeable, skilled, and open to observation of their work and to questioning. From all appearances, they were respected for their skill in making a socially important product. Sellers were largely ignorant of how *bai jiu* was produced. There is a tendency among many rural Chinese, and some urban Chinese, not to ask questions or express curiosity and to accept any available information as adequate. This seems to be a product of recent history and times that discouraged curiosity and special knowledge.

It is regularly reported that unrecorded alcohol has higher ABV. The *bai jiu* sellers we interviewed reported their customers preferred products with higher ABV and were willing to pay a higher price for these products. Higher ABV represents a greater health risk [4, 11].

The data from the interviews indicated that *bai jiu* makers and sellers were part of close social networks in which the alcohol they made and sold is consumed. Specifically, itinerant *bai jiu* makers returned each year to the same villages to make alcohol for long-term customers; therefore their income and reputation depended on their reliably making a safe product. *Bai jiu* makers with permanent production sites are similarly constrained to produce only safe products for their customers, who are also their neighbors.

Data indicated that villagers who sold *bai jiu* in their shops (but who were not themselves *bai jiu* makers) were also constrained from adulterating their product by the necessity of maintaining their good reputation with customers in order to continue selling. None of the *bai jiu* consumers we interviewed mentioned distrust of their local sellers. In most cases, the sellers we interviewed knew and trusted the alcohol makers who supplied their shops with *bai jiu*. We assess the public risk as low because we believe makers and sellers are unlikely to take any action that would undermine their reputation or the trust of their customers.

It is possible that sellers might overstate the ABV levels to their customers, but this practice would reduce risk, not increase it. It is also possible that sellers might stretch their product by adding water, which would lower ABV and increase the chance of introducing microbes via unpurified water to the *bai jiu*, but the typically high ABV and the acidity of the product would destroy most microbes [13].

It was not possible to estimate the number of local *bai jiu* producers, but most villages had at least one *bai jiu* maker, and there were typically several *bai jiu* makers in the towns. The number of itinerant producers is even more difficult to estimate. While each individual *bai jiu* distillery was small, the sheer number of them means that a large, as yet unquantified, amount of *bai jiu* is produced and consumed in this area of rural China. Furthermore, the growing appetite for fruit wine in China suggests rural areas may develop a small, unrecorded fruit wine industry.

### Traditional knowledge

Concerns have been raised about the inadvertent dangers of drinking distilled spirits made by producers who are not skilled at their craft [20]. While the makers we observed and interviewed lacked formally acquired knowledge of chemistry, their final product indicates they follow good practice. For example, without exception the makers we observed knew that the first distillate is poor quality—although they may not have been able to say precisely why it is so. The first distillate is high in acetaldehyde, possibly the one naturally resulting compound in distillation that presents the greatest risk to health. The *bai jiu* makers either discarded the first distillate or diluted it by blending some of it back into the *bai jiu* later in the distillation process. Other risks from distilled unrecorded alcohol result from low-quality production and unhygienic conditions. Reports from other parts of the world suggest this possibility, but we did not observe anything like the conditions described by researchers in Africa, Sri Lanka, or Eastern Europe [8, 21]. The skill of the *bai jiu* makers in this area seemed adequate to prevent most of the dangers associated with unrecorded alcohol.

### Economics

The *bai jiu* makers used grain to produce *bai jiu* and then fed the spent grain to livestock, which farmers considered better feed than unfermented grain. A similar double economy of unrecorded alcohol and livestock feed was found in a study of traditional alcohol production in rural Vietnam [22]. The significant economic benefit of double use is often overlooked in discussions of this type of alcohol. There is also an important nonmonetary value from traditional *bai jiu* production; it confers a special status to the maker. While production of this traditional *bai jiu* is typically not directly taxed, a production license and/or a health certificate is required in some locations, which generates some revenue for local government.

If, as suggested in the WHO *Global Status Reports*, 25 % of alcohol consumed in China is this type, the government is missing considerable alcohol revenues [1]. Commercial alcohol producers and importers find this situation unfair [23]. In China, the government is a significant investor in the commercial alcohol industry. However, imposing and collecting a tax on the many traditional *bai jiu* makers would be costly and difficult. WHO has linked the rise in global per capita alcohol consumption to a rise in advertising, marketing, packaging, and promotion by the commercial alcohol industry [2]. Chinese traditional *bai jiu* is not advertised or marketed in this way, so concerns that marketing increases alcohol consumption are not relevant for this product in this region. The social costs of family problems, the effects on workplace productivity, and the societal costs associated with traffic accidents and the occasional poisoning are not known. Lachenmeier and colleagues have proposed a number of strategies to reduce demand for unrecorded alcohol [24]; however, most of these options themselves carry a cost, which would have to be factored in to policy discussions.

### The future of traditional *bai jiu*

There are questions about the long-term continuation of making, selling, and drinking traditional *bai jiu.* Makers and sellers of bai jiu reported that their customers are mainly older men. Will village-level production disappear as the population of older men declines? Will *bai jiu* be replaced by other unrecorded alcohol products only requiring fermentation? Alternatively, will *bai jiu* be replaced by recorded alcohol products if they become cheaper and the recorded market expands into rural areas? This is entirely possible. Even if traditional *bai jiu* production continues on a small scale, as suggested here, risks are minimal. The challenge for those interested in public health is how to protect these relatively safe small-scale traditional *bai jiu* producers against the increased risks associated with expanded production and enlarged distribution networks that may operate in the relative absence of licensing, supervision, and taxation.

## Conclusion

Traditional *bai jiu* production followed time-tested methods and there was little evidence in the community that suggested hazards other than those associated with any alcohol beverage. Use of traditional *bai jiu* was common and was motivated by the perceptions of better taste, higher quality, lower cost, and the belief that traditional spirits were somehow better than commercial products. Drinking patterns were similar to those found in other studies of alcohol use in China [18]. Daily consumption was common; if a person drank alcohol with meals, it was more common to drink *bai jiu* than to drink recorded alcohols. It was not uncommon for men to drink traditional *bai jiu* alone. Drinking traditional *bai jiu* was an unremarkable part of daily life.

Community members were not concerned about the safety of traditional alcohols. While they identified some alcohol-related problems, they did not associate these problems with traditional *bai jiu*. Estimates of safe drinking levels were reasonable. While the majority of males exceeded these levels, they did not report getting drunk or feeling sick after an episode of overdrinking. Drinking in general was associated with elevating good moods, reducing bad moods, and socializing. Alcohol was thought to alleviate tiredness, and a moderate amount is believed to be good for health.

Taken together, production methods, distribution patterns, consumption patterns did not confirm the levels of risk suggested by others [8], at least for this type of unrecorded alcohol, traditional *bai jiu*, in this part of China. There were no indications that the production methods included significant risk, and the close social networks of sellers and consumers lowered risk of adulteration. Our observations and interviews have led us to the initial conclusion that this type of alcohol, in this region, presents only a low risk to most users. This conclusion is contrary to the conclusions about informal alcohol drawn by alcohol industry-sponsored studies [8, 23] and by public health researchers [25, 26]. Our initial conclusion is similar to the opinion of Lachenmeier and Rehm [9] that the main risk to users is from heavy use combined with the higher alcoholic strength of *bai jiu*—the same risk associated with all types of alcohol beverages.

Makers and sellers indicated that most *bai jiu* drinkers were older men, suggesting *bai jiu* production and use may gradually disappear without control or intervention.

The specific challenge is to preserve the local knowledge and traditions that already mitigate risk from alcohol production and use and find ways to capture their benefits for the future.

### Limitations of this study

The results need to be viewed in light of a number of limitations. Chinese alcohol culture is complicated and varies significantly from region to region [18, 27]. *Bai jiu* production and use also varies from region to region and varies seasonally: The fieldwork was done in the summer and early autumn and only focused on one region. The researchers only observed *bai jiu* makers and sellers who agreed to be observed. One person declined to be observed by the research team. No attempt was made to find producers of other types of alcohol, to select a representative group of *bai jiu* makers, or to determine how many *bai jiu* makers there are in the area. The interview sample was not randomly selected and may not be representative of all drinkers, as some of the interviews were conducted near the premises of traditional *bai jiu* makers or sellers. Nevertheless, the findings of this study were similar to those of a study of traditional alcohol production in rural Vietnam [22], and the patterns of alcohol use described in this sample fall within the patterns described in Millwood et al. [18].

This study lays some groundwork for future studies to confirm, refute, or modify these findings and to guide the development of more structured quantitative studies to better describe traditional *bai jiu* use, its outcomes, and its associated motives.
